# Correction: Stress-induced Cdk5 activity enhances cytoprotective basal autophagy in *Drosophila melanogaster* by phosphorylating acinus at serine^437^

**DOI:** 10.7554/eLife.108230

**Published:** 2025-07-09

**Authors:** Nilay Nandi, Lauren K Tyra, Drew Stenesen, Helmut Krämer

**Keywords:** *D. melanogaster*

Nandi N, Tyra LK, Stenesen D, Krämer H. 2017. Stress-induced Cdk5 activity enhances cytoprotective basal autophagy in Drosophila melanogaster by phosphorylating acinus at serine^437^. *eLife* **6**:e30760. doi: 10.7554/eLife.30760.

Published 11 December 2017

We were notified via PubPeer of errors in Figure 2 and 4.

Figure 2 panel M' was a duplicated image of fat bodies of starved Acn^WT^ larvae stained with lysotracker and DAPI from an identical image previously published in the Journal of Cell Biology 2014;207:253 as Supplement Figure S2A' representing the same genotype and treatment.

The inclusion of this duplications was an unfortunate mistake. In 2011, lysotracker and DAPI stainings were part of the initial characterization of a set of wild type and mutant Acinus transgenes. This included transgenes affecting Caspase cleavage of Acinus (published in the Journal of Cell Biology 2014;207) and those affecting Acn-S437 phosphorylation (published in 2017, eLife 6:e30760) compared to Acn^WT^ controls. We inadvertently selected the same, previously published image of starved Acn^WT^ larval fatbody from the set of micrographs for Figure 2 panel M'. We retrieved an alternative image from the original data and used it to replace the duplicated panel M'.

Figure 2 panel N' represents fat bodies of starved Acn^S437A^ larvae stained with lysotracker (red) and DAPI (blue). The DAPI channel - but not the lysotracker channel - was a duplicated copy of the DAPI channel previously published in the Journal of Cell Biology 2014;207(2) Supplement Figure S2A' representing fed Acn^WT^ fat body.

We suspect that this duplication of the DAPI channel occurred while selecting images from the dedicated folders and assembling them into the collage for an early draft version of Figure 2. As additional data assessing autophagy flux was added to the figure, the group of six lysotracker/DAPI panels from the draft version were turned by 90° from the vertical arrangement to the horizontal shown in the final Figure 2.

We have since retrieved the original image data corresponding to the starved Acinus^S437A^ larval fatbodies and added the correct DAPI channel to the unchanged lysotracker staining.

Figure 4 shows SEM images of fly eyes assessing genetic interactions with Acinus overexpression.

Panel E was a duplication of the SEM image published as Figure 2J in the Journal of Cell Biology 2014;207:253 paper representing the same genotype: flies with the GMR-Gal4 driver but no UAS-transgene.

The inclusion of this duplications was an unfortunate mistake. We have retrieved SEM images of the GMR-Gal4 driver-only eyes taken in parallel to the remaining panels of Figure 4 and replaced the duplicated image in Figure 4 panel E.

Panel 4 P is an internally duplicated image from the same original SEM image used in Figure 6C. Both panels represent eyes of control flies expressing the uas-p35 transgene but no Acinus transgene.

We regret this duplication and have retrieved an alternate, SEM image from this experiment representing the same genotype and replaced the duplicated panel.

The corrected Figure 2 is shown here:

**Figure 1:**
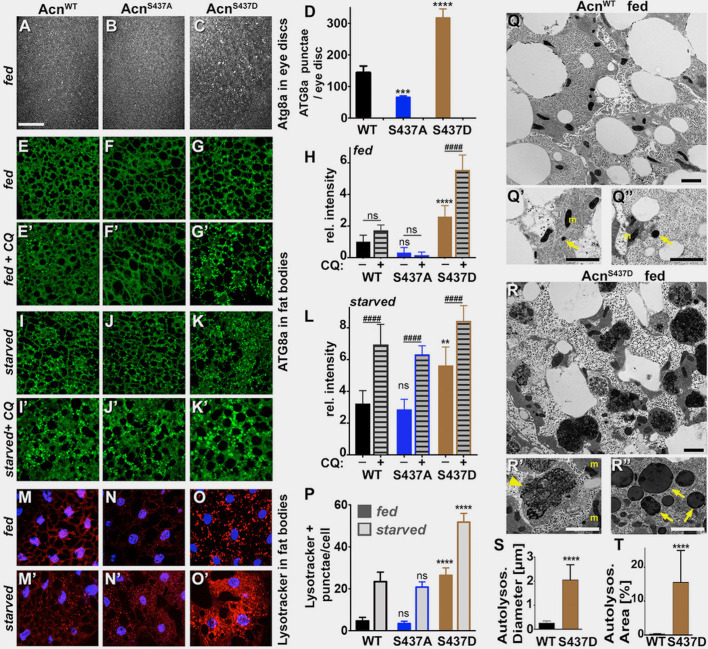


The originally published Figure 2 is shown for reference:

**Figure 2:**
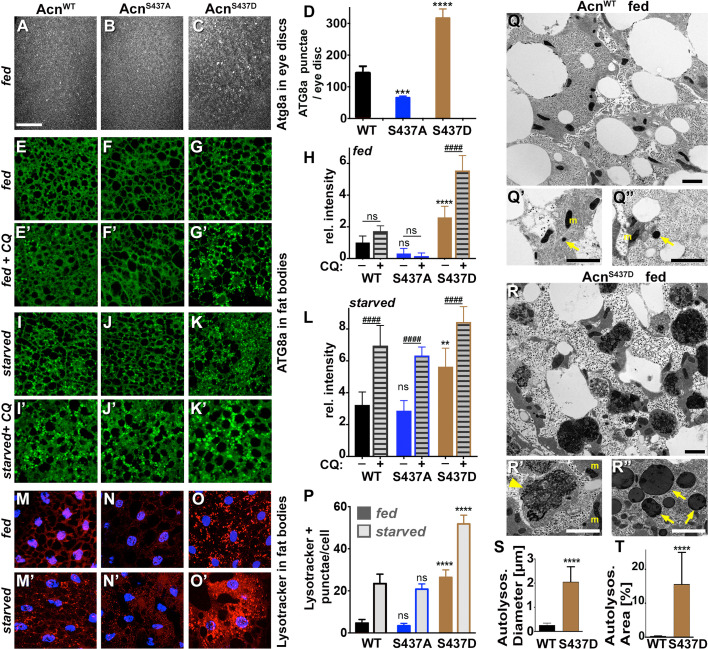


The corrected Figure 4 is shown here:

**Figure 3:**
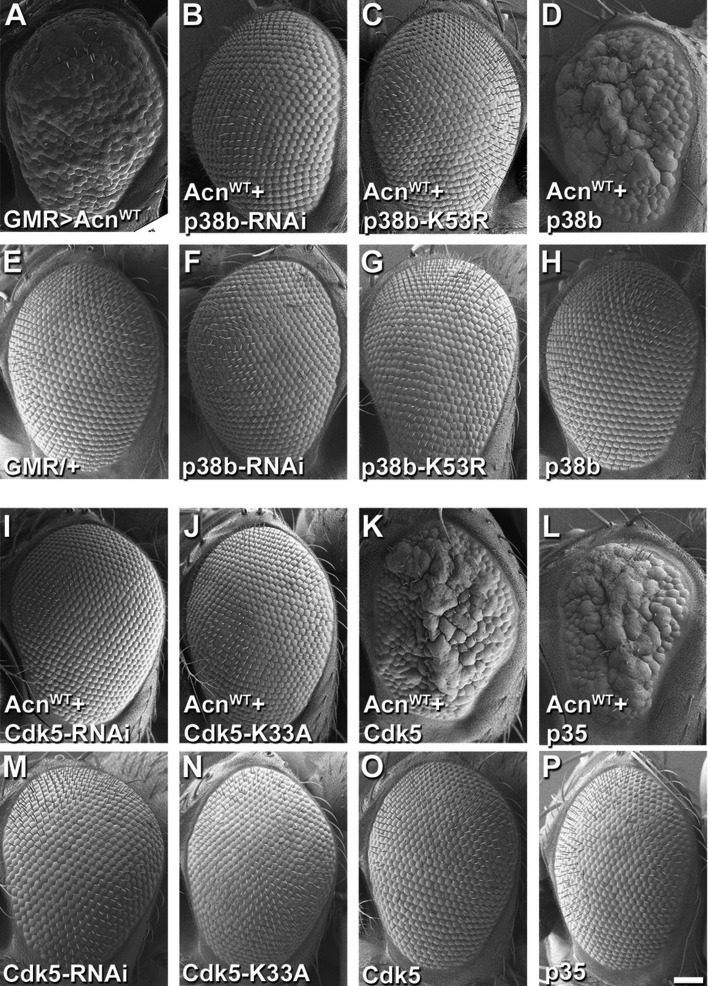


The originally published Figure 4 is shown for reference:

**Figure 4:**
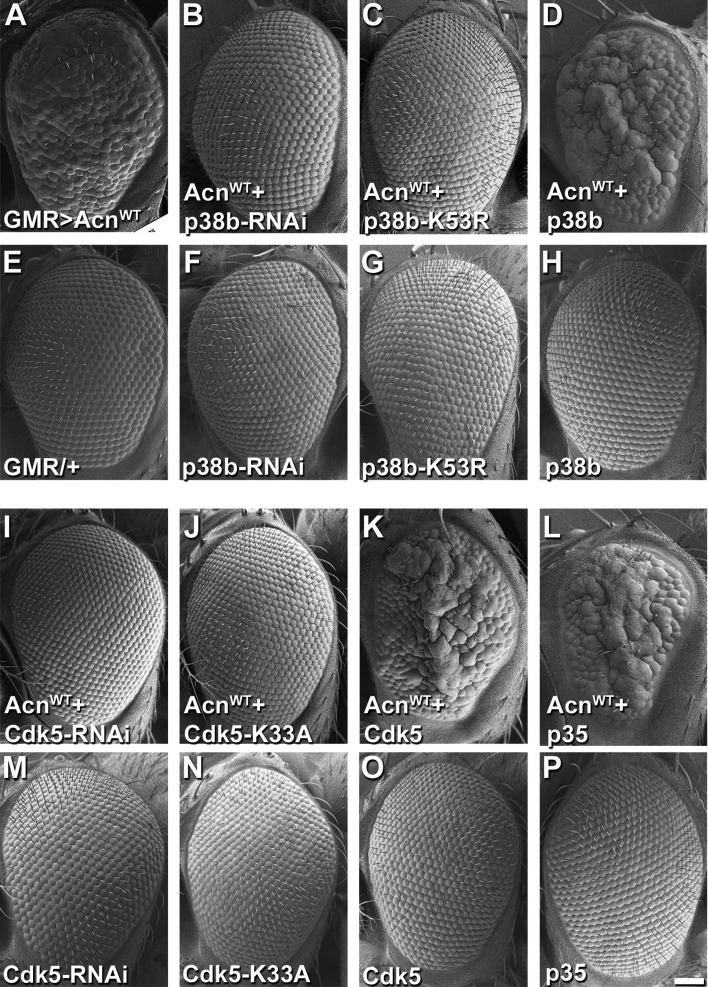


The article has been corrected accordingly.

