# Supplementary material for: Stress-induced Cdk5 activity enhances cytoprotective basal autophagy in Drosophila melanogaster by phosphorylating acinus at serine437
Source: eLife. 2017 Dec 11;6:e30760. doi: 10.7554/eLife.30760 (PMC5760206; doi:10.7554/eLife.30760)
Supplement: Supplementary file 1. — The effect on eye morphology of GMR-Gal4-driven expression of UAS-RNAi transgenes with or without UAS-Acinus was scored and recorded as the number of flies with normal eyes (score = 1), eye with mild errors (score = 2), rough eyes (score = 3) or severely rough eyes (score = 4). The percentage suppression or enhancement was calculated from the average score for each RNAi transgene compared to UAS-Acn in the absence of RNAi transgenes. Positive or negative numbers indicate suppression and enhancement, respectively. Green or red colors highlight UAS-transgenes with more than 50% suppression or enhancement. Numbers in parenthesis indicated stock numbers of the Bloomington Drosophila stock center or, if starting with a ‘V..', from the Vienna Drosophila Resource Center. All flies were raised at 28°C. [file elife-30760-supp1.docx]

| **Supplemental Table I. Suppression of Acn-induced eye roughness by kinase knock down.** | | | | | | | | | | | |
| --- | --- | --- | --- | --- | --- | --- | --- | --- | --- | --- | --- |
|  | **Level of Roughness** | | | | |  | |  | |  |  |
| **GMR-Gal4**  **driven**  **UAS-transgenes** | **Normal** | **Mild  Errors** | **Rough  Eyes** | **Severely  Rough Eyes** | **Average  Score** | | **% ^(1,2)^ Suppression  or Enhancement** | | **n**  **=**  **flies scored** | |  |
| *w*^1118^ (control) | 65 | 0 | 0 | 0 | 1.0 | | NA | | 65 | |  |
| GMR-Gal4 (control) | 57 | 4 | 0 | 0 | 1.1 | | NA | | 61 | |  |
| Acn | 10 | 46 | 16 | 0 | 2.1 | | NA | | 72 | |  |
| bsk RNAi ^(31323)^ + Acn | 17 | 51 | 0 | 10 | 2.0 | | 5 | | 78 | |  |
| bsk RNAi ^(31323)^ | 51 | 29 | 0 | 0 | 1.4 | |  | | 80 | |  |
| rl RNAi ^(36058)^ + Acn | 22 | 47 | 0 | 9 | 1.9 | | 14 | | 78 | |  |
| rl RNAi ^(36058)^ | 61 | 32 | 0 | 0 | 1.3 | |  | | 93 | |  |
| rl RNAi ^(31524)^ + Acn | 21 | 37 | 0 | 12 | 2.0 | | 8 | | 70 | |  |
| rl RNAi (31524) | 59 | 19 | 0 | 0 | 1.2 | |  | | 78 | |  |
| rl RNAi (31387) + Acn | 21 | 40 | 0 | 8 | 1.9 | | 17 | | 69 | |  |
| rl RNAi (31387) | 71 | 25 | 0 | 0 | 1.3 | |  | | 96 | |  |
| rl RNAi (34855) + Acn | 17 | 37 | 0 | 11 | 2.1 | | 0 | | 65 | |  |
| rl RNAi (34855) | 57 | 26 | 0 | 0 | 1.3 | |  | | 83 | |  |
| **p38b RNAi (29405) +Acn** | **42** | **22** | **0** | **0** | 1.3 | | **58** | | 64 | |  |
| p38b RNAi (29405) | 49 | 13 | 0 | 0 | 1.2 | |  | | 62 | |  |
| **p38b RNAi (35252) + Acn** | **32** | **27** | **0** | **0** | 1.5 | | **53** | | 59 | |  |
| p38b RNAi (35252) | 36 | 18 | 0 | 0 | 1.3 | |  | | 54 | |  |
| p38a RNAi (35244) + Acn | 27 | 49 | 0 | 13 | 2.0 | | 9 | | 89 | |  |
| p38a RNAi (35244) | 52 | 29 | 0 | 0 | 1.4 | |  | | 81 | |  |
| p38a RNAi (27316) + Acn | 32 | 51 | 0 | 12 | 1.9 | | 14 | | 95 | |  |
| p38a RNAi (27316) | 49 | 21 | 0 | 0 | 1.3 | |  | | 70 | |  |
| p38a RNAi (34744) + Acn | 22 | 42 | 0 | 9 | 1.9 | | 15 | | 73 | |  |
| p38a RNAi (34744) | 57 | 29 | 0 | 0 | 1.3 | |  | | 86 | |  |
| MAPk-Ak2 RNAi (v3170) + Acn | 11 | 24 | 12 | 2 | 2.1 | | 0 | | 49 | |  |
| MAPk-Ak2 RNAi (v3170) | 47 | 7 | 0 | 0 | 1.1 | |  | | 54 | |  |
| MAPk-Ak2 RNAi (v3171) + Acn | 6 | 32 | 23 | 7 | 2.5 | | -27 | | 68 | |  |
| MAPk-Ak2 RNAi (v3171) | 33 | 8 | 0 | 0 | 1.2 | |  | | 41 | |  |
| S6kII RNAi (v101451) + Acn | 45 | 18 | 14 | 2 | 1.7 | | 33 | | 79 | |  |
| S6kII RNAi (v101451) | 64 | 11 | 0 | 0 | 1.1 | |  | | 75 | |  |
| CaMKII RNAi (v100265) + Acn | 12 | 22 | 7 | 0 | 1.9 | | 18 | | 41 | |  |
| CaMKII RNAi (v100265) | 43 | 7 | 0 | 0 | 1.1 | |  | | 50 | |  |
| Pkc53E RNAi (v27696) + Acn | 22 | 15 | 11 | 2 | 1.9 | | 18 | | 50 | |  |
| Pkc53E RNAi (v27696) | 31 | 6 | 0 | 0 | 1.2 | |  | | 37 | |  |
| **Cdk1 RNAi (v41838) + Acn** | **15** | **3** | **14** | **27** | 2.9 | | **-69** | | 59 | |  |
| Cdk1 RNAi (v41838) | 41 | 14 | 0 | 0 | 1.3 | |  | | 55 | |  |
| **Cdk1 RNAi (v106130) + Acn** | **13** | **4** | **11** | **23** | 2.9 | | **-61** | | 51 | |  |
| Cdk1 RNAi (v106130) | 32 | 10 | 0 | 0 | 1.2 | |  | | 42 | |  |
| Cdk4 RNAi (v40576) + Acn | 12 | 2 | 27 | 0 | 2.4 | | -25 | | 41 | |  |
| Cdk4 RNAi (v40576) | 35 | 8 | 0 | 0 | 1.2 | |  | | 43 | |  |
| Cdk4 RNAi (v40577 )+ Acn | 49 | 3 | 17 | 4 | 1.7 | | 33 | | 73 | |  |
| Cdk4 RNAi (40577) | 39 | 7 | 0 | 0 | 1.2 | |  | | 46 | |  |
| Cdk11 RNAi (v45127) + Acn | 6 | 12 | 4 | 12 | 2.6 | | -17 | | 34 | |  |
| Cdk11 RNAi (v45127) | 37 | 9 | 0 | 0 | 1.2 | |  | | 46 | |  |
| Cdk11 RNAi (v107303) + Acn | 29 | 16 | 19 | 0 | 1.8 | | 25 | | 64 | |  |
| Cdk11 RNAi (v107303) | 45 | 8 | 0 | 0 | 1.2 | |  | | 53 | |  |
| **Cdk5 RNAi (27517) + Acn** | **57** | **7** | **9** | **0** | 1.3 | | **66** | | 73 | |  |
| Cdk5 RNAi (27517) | 72 | 4 | 0 | 0 | 1.1 | |  | | 76 | |  |
| Cdk5 RNAi (35287) + Acn | 32 | 4 | 28 | 0 | 1.9 | | 18 | | 64 | |  |
| Cdk5 RNAi (35287) | 93 | 5 | 0 | 0 | 1.1 | |  | | 98 | |  |
| AMPKα RNAi (25931) + Acn | 4 | 58 | 8 | 0 | 2.1 | | 0 | | 70 | |  |
| AMPKα RNAi (25931) | 56 | 4 | 0 | 0 | 1.1 | |  | | 60 | |  |

All flies were raised at 28°C.

Scores to calculate Average Roughness: normal = 1; mild = 2; rough = 3; strongly rough = 4.

(1) Positive or negative numbers indicate suppression and enhancement, respectively.

(2) Green or red colors highlight UAS-transgenes with more than 50% suppression or enhancement.

(3) Numbers in parenthesis indicated stock numbers of the Bloomington Drosophila stock center or, if starting with a “V..”, from the Vienna Drosophila Resource Center.
