# Supplementary material for: Stress-induced Cdk5 activity enhances cytoprotective basal autophagy in Drosophila melanogaster by phosphorylating acinus at serine437
Source: eLife. 2017 Dec 11;6:e30760. doi: 10.7554/eLife.30760 (PMC5760206; doi:10.7554/eLife.30760)
Supplement: Supplementary file 2. — The effect on eye morphology of GMR-Gal4-driven expression of UAS-transgenes with or without UAS-Acinus was scored and recorded as the number of flies with normal eyes (score = 1), eye with mild errors (score = 2), rough eyes (score = 3) or severely rough eyes (score = 4). The percentage suppression or enhancement was calculated from the average score for each RNAi transgene compared to UAS-Acn in the absence of RNAi transgenes. Positive or negative numbers indicate suppression and enhancement, respectively. Green or red colors highlight UAS-transgenes with more than 50% suppression or enhancement. Numbers in parenthesis indicated stock numbers of the Bloomington Drosophila stock center. All flies were raised at 28°C. [file elife-30760-supp2.docx]

| **Supplemental File 2. Genetic interactions of Acn with p38b and Cdk5/p35 loss and gain of function.** | | | | | | | | | | | |
| --- | --- | --- | --- | --- | --- | --- | --- | --- | --- | --- | --- |
|  | **Level of Roughness** | | | | |  | |  | |  |  |
| **GMR-Gal4**  **driven**  **UAS-transgenes** | **Normal** | **Mild  Errors** | **Rough  Eyes** | **Severely  Rough Eyes** | **Average  Score** | | **% ^(1,2)^**  **Suppression  or Enhancement** | | **n**  **=**  **flies scored** | |  |
| *w*^1118^ | 65 | 0 | 0 | 0 | 1 | | NA | | 65 | |  |
| GMR-Gal4/+ | 57 | 4 | 0 | 0 | 1.1 | | NA | | 61 | |  |
| Acn/+ | 10 | 46 | 16 | 0 | 2.1 | | NA | | 72 | |  |
| p38b + Acn | 0 | 7 | 23 | 30 | 3.4 | | **-107** | | 60 | |  |
| p38b | 51 | 13 | 0 | 0 | 1.2 | |  | | 64 | |  |
| p38b^K53R^ + Acn | 55 | 17 | 0 | 0 | 1.2 | | **79** | | 72 | |  |
| p38b^K53R^ | 37 | 4 | 0 | 0 | 1.1 | |  | | 41 | |  |
| p35 + Acn | 0 | 18 | 12 | 35 | 3.3 | | **-96** | | 65 | |  |
| p35 | 42 | 11 | 0 | 0 | 1.2 | |  | | 53 | |  |
| Cdk5^WT^ + Acn | 0 | 7 | 9 | 11 | 3.1 | | **-92** | | 27 | |  |
| Cdk5^WT^ | 37 | 6 | 0 | 0 | 1.1 | |  | | 43 | |  |
| Cdk5^K33A^ + Acn | 42 | 10 | 3 | 0 | 1.3 | | **74** | | 55 | |  |
| Cdk5^K33A^ | 37 | 4 | 0 | 0 | 1.1 | |  | | 41 | |  |
| p35RNAi(27048) + Acn | 48 | 4 | 0 | 0 | 1.1 | | **91** | | 52 | |  |
| p35RNAi(27048) | 52 | 7 | 0 | 0 | 1.1 | |  | | 59 | |  |
| p35RNAi(27290) + Acn | 45 | 12 | 4 | 0 | 1.3 | | **69** | | 61 | |  |
| p35RNAi(27290) | 47 | 6 | 0 | 0 | 1.1 | |  | | 53 | |  |

All flies were raised at 28°C.

Scores to calculate Average Roughness: normal = 1; mild = 2; rough = 3; strongly rough = 4.

(1) Positive or negative numbers indicate suppression and enhancement, respectively.

(2) Green or red colors highlight UAS-transgenes with more than 50% suppression or enhancement.

(3) Numbers in parenthesis indicated stock numbers of the Bloomington Drosophila stock center
