# Supplementary material for: Stress-induced Cdk5 activity enhances cytoprotective basal autophagy in Drosophila melanogaster by phosphorylating acinus at serine437
Source: eLife. 2017 Dec 11;6:e30760. doi: 10.7554/eLife.30760 (PMC5760206; doi:10.7554/eLife.30760)
Supplement: Supplementary file 3. — Relevant genotypes are listed for all Figures. [file elife-30760-supp3.docx]

**Supplemental Table 3: Genotypes of Flies Used for Each Figure**

| Figure 1 | Genotype |
| --- | --- |
| 1 C | *w^*^* ; *acn^1^* / ubi-GFP *acn^27^* ubi-GFP; P[*w*^+^, *acn*P-Myc-Acn^WT^]^96F3^ |
| 1 D | *w^*^* ; *acn^1^* / ubi-GFP *acn^27^* ubi-GFP; P[*w*^+^, *acn*P-Myc-Acn^S437A^]^96F3^ |
| 1 E | *w^*^* ; *acn^1^* / ubi-GFP *acn^27^* ubi-GFP; P[*w*^+^, *acn*P-Myc-Acn^WT^]^96F3^ |
| 1 F | *w^*^* ; *acn^1^* / ubi-GFP *acn^27^* ubi-GFP; P[*w*^+^, *acn*P-Myc-Acn^WT^]^96F3^ |
| 1 G | *w^*^* ; *acn^1^* / ubi-GFP *acn^27^* ubi-GFP; P[*w*^+^, *acn*P-Myc-Acn^S437A^]^96F3^ |
| 1 H | *w^*^* ; *acn^1^* / ubi-GFP *acn^27^* ubi-GFP; P[*w*^+^, *acn*P-Myc-Acn^S437D^]^96F3^ |
| 1 I, J, K | *w^*^* ; *acn^1^* / ubi-GFP *acn^27^* ubi-GFP; P[*w*^+^, *acn*P-Myc-Acn^WT^]^96F3^  *w^*^* ; *acn^1^* / ubi-GFP *acn^27^* ubi-GFP; P[*w*^+^, *acn*P-Myc-Acn^S437A^]^96F3^  *w^*^* ; *acn^1^* / ubi-GFP *acn^27^* ubi-GFP; P[*w*^+^, *acn*P-Myc-Acn^S437D^]^96F3^ |

| Fig1-Fig Suppl 1 | Genotype |
| --- | --- |
| 1-1 A | *w^*^* ; *acn^1^* / ubi-GFP *acn^27^* ubi-GFP; P[*w*^+^, *acn*P-Myc-Acn^WT^]^96F3^ |
| 1-1 B | *w^*^* ; *acn^1^* / ubi-GFP *acn^27^* ubi-GFP; P[*w*^+^, *acn*P-Myc-Acn^S437A^]^96F3^ |
| 1-1 C | *w^*^* ; *acn^1^* / ubi-GFP *acn^27^* ubi-GFP; P[*w*^+^, *acn*P-Myc-Acn^S437D^]^96F3^ |

| Figure 2 | Genotype |
| --- | --- |
| 2 A | *w^*^* ; *acn^1^* / ubi-GFP *acn^27^* ubi-GFP; P[*w*^+^, *acn*P-Myc-Acn^WT^]^96F3^ |
| 2 B | *w^*^* ; *acn^1^* / ubi-GFP *acn^27^* ubi-GFP; P[*w*^+^, *acn*P-Myc-Acn^S437A^]^96F3^ |
| 2 C | *w^*^* ; *acn^1^* / ubi-GFP *acn^27^* ubi-GFP; P[*w*^+^, *acn*P-Myc-Acn^S437D^]^96F3^ |
| 2 D | *w^*^* ; *acn^1^* / ubi-GFP *acn^27^* ubi-GFP; P[*w*^+^, *acn*P-Myc-Acn^WT^]^96F3^  *w^*^* ; *acn^1^* / ubi-GFP *acn^27^* ubi-GFP; P[*w*^+^, *acn*P-Myc-Acn^S437A^]^96F3^  *w^*^* ; *acn^1^* / ubi-GFP *acn^27^* ubi-GFP; P[*w*^+^, *acn*P-Myc-Acn^S437D^]^96F3^ |
| 2 E | *w^*^* ; *acn^1^* / ubi-GFP *acn^27^* ubi-GFP; P[*w*^+^, *acn*P-Myc-Acn^WT^]^96F3^ |
| 2 F | *w^*^* ; *acn^1^* / ubi-GFP *acn^27^* ubi-GFP; P[*w*^+^, *acn*P-Myc-Acn^S437A^]^96F3^ |
| 2 G | *w^*^* ; *acn^1^* / ubi-GFP *acn^27^* ubi-GFP; P[*w*^+^, *acn*P-Myc-Acn^S437D^]^96F3^ |
| 2 H and  suppl 2- A | *w^*^* ; *acn^1^* / ubi-GFP *acn^27^* ubi-GFP; P[*w*^+^, *acn*P-Myc-Acn^WT^]^96F3^  *w^*^* ; *acn^1^* / ubi-GFP *acn^27^* ubi-GFP; P[*w*^+^, *acn*P-Myc-Acn^S437A^]^96F3^  *w^*^* ; *acn^1^* / ubi-GFP *acn^27^* ubi-GFP; P[*w*^+^, *acn*P-Myc-Acn^S437D^]^96F3^ |
| 2 I | *w^*^* ; *acn^1^* / ubi-GFP *acn^27^* ubi-GFP; P[*w*^+^, *acn*P-Myc-Acn^WT^]^96F3^ |
| 2 J | *w^*^* ; *acn^1^* / ubi-GFP *acn^27^* ubi-GFP; P[*w*^+^, *acn*P-Myc-Acn^S437A^]^96F3^ |
| 2 K | *w^*^* ; *acn^1^* / ubi-GFP *acn^27^* ubi-GFP; P[*w*^+^, *acn*P-Myc-Acn^S437D^]^96F3^ |
| 2 L and suppl 2-B | *w^*^* ; *acn^1^* / ubi-GFP *acn^27^* ubi-GFP; P[*w*^+^, *acn*P-Myc-Acn^WT^]^96F3^  *w^*^* ; *acn^1^* / ubi-GFP *acn^27^* ubi-GFP; P[*w*^+^, *acn*P-Myc-Acn^S437A^]^96F3^  *w^*^* ; *acn^1^* / ubi-GFP *acn^27^* ubi-GFP; P[*w*^+^, *acn*P-Myc-Acn^S437D^]^96F3^ |
| 2 M | *w^*^* ; *acn^1^* / ubi-GFP *acn^27^* ubi-GFP; P[*w*^+^, *acn*P-Myc-Acn^WT^]^96F3^ |
| 2 N | *w^*^* ; *acn^1^* / ubi-GFP *acn^27^* ubi-GFP; P[*w*^+^, *acn*P-Myc-Acn^S437A^]^96F3^ |
| 2 O | *w^*^* ; *acn^1^* / ubi-GFP *acn^27^* ubi-GFP; P[*w*^+^, *acn*P-Myc-Acn^S437D^]^96F3^ |
| 2 P | *w^*^* ; *acn^1^* / ubi-GFP *acn^27^* ubi-GFP; P[*w*^+^, *acn*P-Myc-Acn^WT^]^96F3^  *w^*^* ; *acn^1^* / ubi-GFP *acn^27^* ubi-GFP; P[*w*^+^, *acn*P-Myc-Acn^S437A^]^96F3^  *w^*^* ; *acn^1^* / ubi-GFP *acn^27^* ubi-GFP; P[*w*^+^, *acn*P-Myc-Acn^S437D^]^96F3^ |
| 2 Q | *w^*^* ; *acn^1^* / ubi-GFP *acn^27^* ubi-GFP; P[*w*^+^, *acn*P-Myc-Acn^WT^]^96F3^ |
| 2 R | *w^*^* ; *acn^1^* / ubi-GFP *acn^27^* ubi-GFP; P[*w*^+^, *acn*P-Myc-Acn^S437D^]^96F3^ |
| 2 S, T | *w^*^* ; *acn^1^* / ubi-GFP *acn^27^* ubi-GFP; P[*w*^+^, *acn*P-Myc-Acn^WT^]^96F3^  *w^*^* ; *acn^1^* / ubi-GFP *acn^27^* ubi-GFP; P[*w*^+^, *acn*P-Myc-Acn^S437D^]^96F3^ |

| Figure 3 | Genotype |
| --- | --- |
| 3 A, B | *w^*^* ; *acn^27^*; P[*w*^+^, *acn*P-Myc-Acn^WT^]^96F3^ / +  *w^*^* ; *acn^27^*; P[*w*^+^, *acn*P-Myc-Acn^S437A^]^96F3^ / +  *w^*^* ; *acn^27^* ; P[*w*^+^, *acn*P-Myc-Acn^S437D^]^96F3^ / + |

| Figure 4 | Genotype |
| --- | --- |
| 4 A | *w^*^* ; GMR*-*Gal4, P[*w*^+^, UAS-Acn^WT^] / CyO; *Sb*/TM6b |
| 4 B | *w^*^* ; GMR*-*Gal4, P[*w*^+^, UAS-Acn^WT^] / +; *Sb* / UAS-p38b RNAi (BS29405)  *equivalent results, data not shown:*  p38b RNAi line: BS35252 |
| 4 C | *w^*^* ; GMR*-*Gal4, P[*w*^+^, UAS-Acn^WT^] / UAS-p38b K53R; *Sb* / + |
| 4 D | *w^*^* / UAS-p38b ; GMR*-*Gal4, P[*w*^+^, UAS-Acn^WT^] / +; *Sb* / + |
| 4 E | *w^*^* ; GMR-Gal4 / +; *Sb* / + |
| 4 F | *w^*^* ; GMR-Gal4 / +; *Sb* / UAS-p38b RNAi (BS29405) |
| 4 G | *w^*^* ; GMR-Gal4 / UAS-p38b^K53R^; *Sb* / + |
| 4 H | *w^*^* / UAS-p38b ; GMR*-*Gal4 / +; *Sb* / + |
| 4 I | *w^*^* ; GMR*-*Gal4, P[*w*^+^, UAS-Acn^WT^] / +; *Sb* / UAS-Cdk5 RNAi (BS27517) |
| 4 J | *w^*^* ; GMR*-*Gal4, P[*w*^+^, UAS-Acn^WT^] / UAS-Cdk5^K33A^; *Sb* / + |
| 4 K | *w^*^* ; GMR*-*Gal4, P[*w*^+^, UAS-Acn^WT^] / UAS-Cdk5; *Sb* / + |
| 4 L | *w^*^* ; GMR*-*Gal4, P[*w*^+^, UAS-Acn^WT^] / +; *Sb* / UAS-p35 |
| 4 M | *w^*^* ; GMR*-*Gal4 / +; *Sb* / UAS-Cdk5 RNAi (BS27517) |
| 4 N | *w^*^* ; GMR*-*Gal4 / UAS-Cdk5^K33A^; *Sb* / + |
| 4 O | *w^*^* ; GMR*-*Gal4 / UAS-Cdk5; *Sb* / + |
| 4 P | *w^*^* ; GMR*-*Gal4 / +; *Sb* / UAS-p35 |

| Fig 4-Fig Suppl 1 | Genotype |
| --- | --- |
|  | OreR |
|  | *w^*^* ; *Sp* or CyO /+; da-Gal4/ UAS-p38b RNAi (BS29405) |
|  | *w^*^* ; *Sp* or CyO /+; da-Gal4/ UAS-p38b RNAi (BS35252) |
|  | OreR |
|  | *w^*^* ; *Sp* or CyO /+; da-Gal4/ UAS-Cdk5 RNAi (BS27517) |
|  | OreR |
|  | *w^*^* ; + / +; arm-Gal4 / UAS-p35 RNAi (BS27048) |
|  | *w^*^* ; + / +; arm-Gal4 / UAS-p35 RNAi (BS27290) |

| Fig 4-Fig Suppl 2 | Genotype |
| --- | --- |
| 4-1 A | *w^*^* ; GMR*-*Gal4 / UAS-FLAG-Cdk5^K33A^; *Sb* / + |
| 4-1 B | *w^*^* ; GMR-Gal4 / UAS-FLAG-p38b^K53R^; *Sb* / + |

| Figure 5 | Genotype |
| --- | --- |
| 5 A, B, C | OreR |
| 5 D | *w^*^* ; *Sp* or CyO /+; da-Gal4/ UAS-Cdk5 RNAi (BS27517) |
| 5 E, F | *w^*^* ; +/+; arm-Gal4/ UAS-Cdk5 RNAi (BS27517) |
| 5 G | *w^*^* ; *cdk5 null^#12^* / Df(2R)ED2426 |
| 5 H | *w^*^* ; *p35^20c^* |
| 5 I | *w^*^* ; + / +; arm-Gal4 / UAS-p35 RNAi (BS27048) |
| 5 J | *w^*^* ; *cdk5 null^#12^* / Df(2R)ED2426; Cdk5wt^#71^ |
| 5 K | *w^*^* ; + / +; arm-Gal4 / UAS-p35 |
| 5 L | *w^*^* ; *p38Kb^Δ45^* |

| Figure 6 | Genotype |
| --- | --- |
| 6 A | *w^*^* ; GMR*-*Gal4, P[*w*^+^, pAttb-UAS-Myc-Acn^WT^]^43A1^ / +; *Sb* / + |
| 6 B | *w^*^* ; GMR*-*Gal4, P[*w*^+^, pAttb-UAS-Myc-Acn^S437A^]^43A1^ / +; *Sb* / + |
| 6 C | *w^*^* ; GMR*-*Gal4 / +; *Sb* / UAS-p35 |
| 6 D | *w^*^* / UAS-p38b ; GMR*-*Gal4 / +; *Sb* / + |
| 6 E | *w^*^* ; GMR*-*Gal4, P[*w*^+^, pAttb-UAS-Myc-Acn^WT^]^43A1^ / +; *Sb* / UAS-p35 |
| 6 F | *w^*^* ; GMR*-*Gal4, P[*w*^+^, pAttb-UAS-Myc-Acn^S437A^]^43A1^ / +; *Sb* / UAS-p35 |
| 6 G | *w^*^* / UAS-p38b ; GMR*-*Gal4, P[*w*^+^, pAttb-UAS-Myc-Acn^WT^]^43A1^ / +; *Sb* / + |
| 6 H | *w^*^* / UAS-p38b ; GMR*-*Gal4, P[*w*^+^, pAttb-UAS-Myc-Acn^S437A^]^43A1^ / +; *Sb* / + |
| 6 I | *w^*^* ; GMR*-*Gal4, P[*w*^+^, pAttb-UAS-Myc-Acn^WT^]^43A1^ / +; *Sb* / +  *w^*^* ; GMR*-*Gal4, P[*w*^+^, pAttb-UAS-Myc-Acn^S437A^]^43A1^ / +; *Sb* / +  *w^*^* / UAS-p38b ; GMR*-*Gal4 / +; *Sb* / +  *w^*^* / UAS-p38b ; GMR*-*Gal4, P[*w*^+^, pAttb-UAS-Myc-Acn^WT^]^43A1^ / +; *Sb* / +  *w^*^* / UAS-p38b ; GMR*-*Gal4, P[*w*^+^, pAttb-UAS-Myc-Acn^S437A^]^43A1^ / +; *Sb* / +  *w^*^* ; GMR*-*Gal4 / +; *Sb* / UAS-p35  *w^*^* ; GMR*-*Gal4, P[*w*^+^, pAttb-UAS-Myc-Acn^WT^]^43A1^ / +; *Sb* / UAS-p35  *w^*^* ; GMR*-*Gal4, P[*w*^+^, pAttb-UAS-Myc-Acn^S437A^]^43A1^ / +; *Sb* / UAS-p35 |

| Figure 7 | Genotype |
| --- | --- |
| 7 A, F | OreR |
| 7 B, G | *w^*^* ; *p35^20c^* |
| 7 C, H | *w^*^* ; *p35^20c^* ; P[*w*^+^, *acn*P-Myc-Acn^WT^]^96F3^ / + |
| 7 D, I | *w^*^* ; *p35^20c^* ; P[*w*^+^, *acn*P-Myc-Acn^S437A^]^96F3^ / + |
| 7 E, J | *w^*^* ; *p35^20c^* ; P[*w*^+^, *acn*P-Myc-Acn^S437D^]^96F3^ / + |
| 7 K, L, M | OreR  *w^*^* ; *p35^20c^*  *w^*^* ; *p35^20c^* ; P[*w*^+^, *acn*P-Myc-Acn^WT^]^96F3^ / +  *w^*^* ; *p35^20c^* ; P[*w*^+^, *acn*P-Myc-Acn^S437A^]^96F3^ / +  *w^*^* ; *p35^20c^* ; P[*w*^+^, *acn*P-Myc-Acn^S437D^]^96F3^ / + |

| Figure 8 | Genotype |
| --- | --- |
| 8 A | *w^*^* ; GMR*-*Gal4 / +;  *Sb* / + |
| 8 B | *w^*^* ; GMR*-*Gal4 / +; UAS-Htt.Q93 / *Sb* |
| 8 C | *w^*^* ; GMR*-*Gal4 / +; UAS-Htt.Q93 / UAS-p35 RNAi (BS27048) |
| 8 D | *w^1118^* ; wg^sp-1^ / CyO; GMR-Gal4-Htt.Q120 / TM6b, Tb^1^ |
| 8 E | *w^1118^* ;  *p35^20c^*; GMR-Gal4-Htt.Q120 / TM6b, Tb^1^ |
| 8 F | *w^*^* ; GMR*-*Gal4 / +;  *Sb* / +  *w^*^* ; GMR*-*Gal4 / +; UAS-Htt.Q93 / *Sb*  *w^*^* ; GMR*-*Gal4 / +; UAS-Htt.Q93 / UAS-p35 RNAi (BS27048)  *w^1118^* ; wg^sp-1^ / CyO; GMR-Gal4-Htt.Q120 / TM6b, Tb^1^  *w^1118^* ;  *p35^20c^*; GMR-Gal4-Htt.Q120 / TM6b, Tb^1^ |
| 8 G | *w^*^* ; GMR*-*Gal4 / +;  *Sb* / + |
| 8 H | *w^1118^*; wg^sp-1^ / CyO; GMR-Gal4-Htt.Q120 / P[*w*^+^, *acn*P-Myc-Acn^WT^] |
| 8 I | *w^1118^*; wg^sp-1^ / CyO; GMR-Gal4-Htt.Q120 / P[*w*^+^, *acn*P-Myc-Acn^S437A^] |
| 8 J | *w^1118^*; wg^sp-1^ / CyO; GMR-Gal4-Htt.Q120 / P[*w*^+^, *acn*P-Myc-Acn^S437D^] |
| 8 H | *w^1118^* ;  *p35^20c^*; GMR-Gal4-Htt.Q120 / TM6b, Tb^1^ |
| 8 L, M | *w^*^* ; GMR*-*Gal4 / +;  *Sb* / +  *w^1118^*; wg^sp-1^ / CyO; GMR-Gal4-Htt.Q120 / P[*w*^+^, *acn*P-Myc-Acn^WT^]  *w^1118^*; wg^sp-1^ / CyO; GMR-Gal4-Htt.Q120 / P[*w*^+^, *acn*P-Myc-Acn^S437A^]  *w^1118^*; wg^sp-1^ / CyO; GMR-Gal4-Htt.Q120 / P[*w*^+^, *acn*P-Myc-Acn^S437D^]  *w^1118^* ;  *p35^20c^*; GMR-Gal4-Htt.Q120 / TM6b, Tb^1^ |

| Fig 9 | Genotype |
| --- | --- |
| 9 A | *w^*^* ; GMR*-*Gal4 / +;  *Sb* / + |
| 9 B | *w^*^* ; GMR*-*Gal4 / UAS-hSap\MJD.tr-Q78; *Sb / +* |
| 9 C | *w^*^* ; GMR*-*Gal4 / +; *Sb /*  UAS-hSap\ATX1.Q82 |
| 9 D | *w^*^* ; GMR*-*Gal4 / UAS-APP. Aβ 1-42; *Sb / +* |
| 9 E | *w^*^* ; GMR*-*Gal4 / UAS-SNCA.J; *Sb / +* |
| 9 F | *w^*^* ; GMR*-*Gal4 / +; *Sb /*  UAS-hSOD1 |
| 9 G | *w^*^* ; GMR*-*Gal4 / +;  *Sb* / +  *w^*^* ; GMR*-*Gal4 / UAS-hSap\MJD.tr-Q78; *Sb / +*  *w^*^* ; GMR*-*Gal4 / +; *Sb /*  UAS-hSap\ATX1.Q82  *w^*^* ; GMR*-*Gal4 / UAS-APP. Aβ 1-42; *Sb / +*  *w^*^* ; GMR*-*Gal4 / UAS-SNCA.J; *Sb / +*  *w^*^* ; GMR*-*Gal4 / +; *Sb /*  UAS-hSOD1 |
